# Anomalous displacement reaction for synthesizing above-room-temperature and air-stable vdW ferromagnet PtTe_2_Ge_1/3_

**DOI:** 10.1093/nsr/nwac173

**Published:** 2022-08-18

**Authors:** Wenxuan Zhu, Cheng Song, Qian Wang, Hua Bai, Siqi Yin, Feng Pan

**Affiliations:** Key Laboratory of Advanced Materials, School of Materials Science and Engineering, Beijing Innovation Center for Future Chips, Tsinghua University, Beijing 100084, China; Key Laboratory of Advanced Materials, School of Materials Science and Engineering, Beijing Innovation Center for Future Chips, Tsinghua University, Beijing 100084, China; Key Laboratory of Advanced Materials, School of Materials Science and Engineering, Beijing Innovation Center for Future Chips, Tsinghua University, Beijing 100084, China; Key Laboratory of Advanced Materials, School of Materials Science and Engineering, Beijing Innovation Center for Future Chips, Tsinghua University, Beijing 100084, China; Key Laboratory of Advanced Materials, School of Materials Science and Engineering, Beijing Innovation Center for Future Chips, Tsinghua University, Beijing 100084, China; Key Laboratory of Advanced Materials, School of Materials Science and Engineering, Beijing Innovation Center for Future Chips, Tsinghua University, Beijing 100084, China

**Keywords:** vdW magnets, room-temperature ferromagnetism, air stability, HSAB, PtTe_2_Ge_1/3_

## Abstract

Emerging van der Waals (vdW) magnets provide a paradise for the exploration of magnetism in the ultimate two-dimensional (2D) limit, and the construction of integrated spintronic devices, and have become a research frontier in the field of low-dimensional materials. To date, prototypical vdW magnets based on metals of the first transition series (e.g. V, Cr, Mn and Fe) and chalcogen elements suffer from rapid oxidation restricted by the Hard-Soft-Acid-Base principle, as well as low Curie temperatures (*T*_C_), which has become a generally admitted challenge in 2D spintronics. Here, starting from air-unstable Cr_2_Ge_2_Te_6_ vdW thin flakes, we synthesize Ge-embedded PtTe_2_ (namely PtTe_2_Ge_1/3_) with superior air stability, through the displacement reaction in the Cr_2_Ge_2_Te_6_/Pt bilayer. In this process, the anomalous substitution of Cr with Pt in the thermal diffusion is inverse to the metal activity order, which can be attributed to the compatibility between soft-acid (Pt) and soft-base (Te) elements. Meanwhile, the layered uniform insertion of Ge unbalances Pt–Te bonds and introduces long-range ordered ferromagnetism with perpendicular magnetic anisotropy and a Curie temperature above room temperature. Our work demonstrates the anti-metal-activity-order reaction tendency unique in 2D transition-metal magnets and boosts progress towards practical 2D spintronics.

## Teaser

A novel van der Waals ferromagnet PtTe_2_Ge_1/3_ with above room-temperature Curie temperature and high air stability was synthesized through an anti-metal-activity-order displacement reaction in Pt/Cr_2_Ge_2_Te_6_ heterostructure.

## INTRODUCTION

Since their discovery, two-dimensional (2D) van der Waals (vdW) crystals have opened up exciting prospects for next-generation integrated circuits, photoelectronics and topological electronics [1–6]. With both exchange interaction and magnetic anisotropy, magnetism is also able to survive in 2D systems [7,8], which enriches our fundamental understanding of magnetism and adds a different functional dimension to 2D materials, creating potential applications in magnetic memories and magnetoresistance sensors [9–11]. Prototypical spintronic devices based on Cr_2_Ge_2_Te_6_ (CGT) [12–17], CrI_3_ [18–21] and Fe_3_GeTe_2_ [22–25] have been realized experimentally, and exhibit fine interfaces that create unique interfacial effects in magnetic heterostructures [25,26], and distinct dimensions inherent to 2D materials, such as the stacking order [27,28], for the regulation of magnetic properties. Up to now, the low Curie temperature (*T*_C_) and the rapid degradation of magnetism in air have seriously impeded the practical applications of vdW magnets, and this has become the research focus of 2D materials.

Great efforts have been made with regard to the challenge of raising the *T*_C_ of vdW magnets. 1*T* phase transition-metal di-chalcogenides (TMDs), such as MnSe_2_ [29], VSe_2_ [30] (in doubt [31]) and CrTe_2_ [32,33], were found to show the room-temperature *T*_C_, but possess rather weak stability, bringing about difficulties in synthesis and device fabrication. The ionic gating [22], change of composition [34] and proximity effect from a strong spin-orbit coupling layer (such as Bi_2_Te_3_) [35] were proven to effectively elevate the *T*_C_ of vdW Fe_3_GeTe_2_ thin flakes. Although the results of raising the *T*_C_ to room temperature are inspiring, the ionic gating leads to weak magnetization with negligible remanence (<10%), and the sensitive topological insulator is still a severe obstacle. Most importantly, the above methods are still unable to solve the problem of the magnetism degradation of vdW thin flakes in air and have low compatibility with the Si-based CMOS (complementary metal-oxide-semiconductor) technique. Therefore, vdW magnetic thin films with combined room-temperature *T*_C_ and air-stable ferromagnetism remain unrealized and vigorously pursued.

We comprehend that the air stability of conventional vdW magnets composed of hard acids (3*d* transition metals, e.g. Cr^3+^, Fe^3+^ and Mn^4+^) and soft bases (S^2–^, Te^2–^, I^–^) is naturally restricted by the Hard-Soft-Acid-Base (HSAB) principle [36], in which the incompatibility between the cations and anions results in rapid oxidation even in comparably thick samples (tens of nanometers) [17,33], thus a protective layer is required in devices [25,33]. In this work, starting from CGT, we prepared layered uniformly Ge-embedded PtTe_2_ (i.e. PtTe_2_Ge_1/3_) by substituting Cr with Pt in the precursor framework CGT via thermal diffusion. Such an anti-metal-activity-order displacement reaction is driven by the strong affinity between Pt and Te from the HSAB principle, which reduces the total energy. The layered Ge atoms from original CGT are the natural source of uniform doping in PtTe_2_, in which 5*d* transition metal Pt is close to the Stoner criterion and has the opportunity to contribute to the ferromagnetism. Thus, long-range ordered ferromagnetism is realized. Consequently, the novel vdW magnet, PtTe_2_Ge_1/3_, shows ferromagnetism above room temperature, which is associated with robust perpendicular magnetic anisotropy (PMA) (remanence ∼80% at 300 K) and high air stability in thin flakes with the thickness down to 10 nm. The present findings will provide a unique synthesis pathway for vdW magnets and advance practical 2D spintronics.

## RESULTS

**Figure 1.**
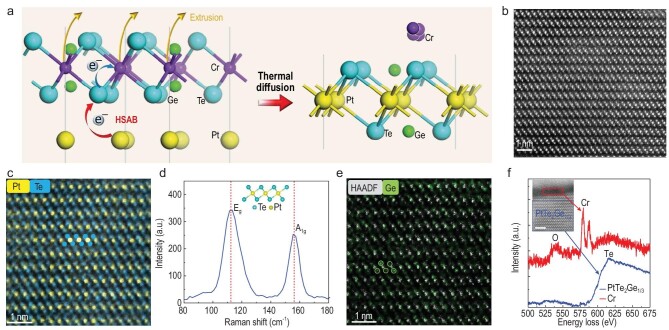
Schematic of the reaction and structural characterizations of PtTe_2_Ge_1/3_. (a) Schematic of the phase transition dominated by the HSAB principle during the thermal diffusion reaction. (b) HAADF-STEM cross-sectional image of the reacted *t* = 14 nm sample. (c) Atomic-resolution EDS mapping of Pt and Te elements. (d) Raman spectrum of the obtained sample. The inset shows the lattice structure of PtTe_2_. (e) EDS mapping of the Ge element. (f) EELS spectra of vdW PtTe_2_Ge_1/3_ (blue square, obtained from the integrated spectrum of EELS area mapping) and the substituted Cr on the surface (red square). The double peaks around 584 eV, and single peaks around 532 eV and 615 eV, are features of Cr, O and Te, respectively. The inset shows the low-resolution image. The crystallized Cr is highlighted by a red square. Scale bar: 2 nm. The top bright layer in the image is the protective layer (platinum) for the preparation of STEM samples.

Generally, vdW non-magnets consisting of soft-acid (Pt, W) and soft-base elements (Se, Te), such as PtTe_2_, are hardly oxidized in air, except in a 2D limit with quite a large specific surface area. Despite this, without effective doping, PtTe_2_ itself is non-magnetic or merely exhibits short-range magnetism from intrinsic defects at extremely low temperatures (below 40 K) [37,38]. Therefore, introducing layered ordered doping into PtTe_2_ with non-magnetic atoms is a promising way to induce air-stable and long-range ordered ferromagnetism above room temperature. However, inducing large amount of doping by conventional methods of film growth (such as molecular beam epitaxy and chemical vapor deposition) tends to form segregated doping or second phases, unfavorable for the emergence of stable structures and robust ferromagnetism. Enlightened by the ordered arrangement of Ge atoms in CGT, we proposed a thermal diffusion reaction in which Cr is replaced with Pt while Ge ordering is maintained, the schematic of which is shown in Fig. 1a.

\begin{eqnarray*} &&{\rm{Pt}}\, + \,{\rm{C}}{{\rm{r}}}_2{\rm{G}}{{\rm{e}}}_{\rm{2}}{\rm{T}}{{\rm{e}}}_{\rm{6}} \to {\rm{PtT}}{{\rm{e}}}_2\!\left( {{\rm{Ge}}} \right)\!x\\ &&\qquad + \,{\rm{Cr}}\,{\rm{ + }}\,{\rm{by\!}}-{\rm{\!product}} \end{eqnarray*}

This anomalous substitution can be driven by the stronger affinity of Pt (soft acid) and Te (soft base) according to the HSAB theory, in which Te 5*p* electrons disperse to Pt *d*^2^*sp*^3^ hybridized orbitals, effectively preventing the oxidization of anions.

Based on this design, a 6 nm Pt film was initially deposited on the Si/SiO_2_ substrate, followed by the exfoliation of vdW CGT thin flakes on it (Fig. S1). Then the Pt/CGT heterostructure was heated at 400°C in a vacuum for 4 hours in order to stimulate a sufficient thermal diffusion reaction between Pt and CGT. We then used high-angle annular dark-field scanning transmission electron microscopy (HAADF-STEM) imaging to investigate the structure after the reaction. A typical HAADF-STEM cross-sectional image of the thickness *t* = 14 nm sample is shown in Fig. 1b. Remarkably, a uniform layered structure with vdW gaps exists in the sample, which possesses a different structure from CGT, demonstrating the formation of a different 2D material. The atomic energy dispersive spectrometer (EDS) mapping of the cross section discloses that Pt and Te atoms construct the crystal lattice (Fig. 1c), giving rise to the PtTe_2_ framework [39], as highlighted in the inset. The Raman spectrum shown in Fig. 1d)exhibits the double characteristic modes of PtTe_2_, E_g_ and A_1g_, respectively arising from the in-plane vibration and out-of-plane vibration of the Pt–Te bond. Compared with the double characteristic modes of the Cr–Te bond in CGT (Fig. S1), the E_g_ modes of Pt–Te in PtTe_2_ and Cr–Te in CGT are naturally close to each other [12,39]. Nevertheless, the complete disappearance of the A_1__g_ mode from Cr–Te and its full replacement by Pt–Te demonstrate the absence of Cr–Te bonds from CGT. This observation supports the complete phase transition from CGT to PtTe_2_, whose lattice structure is shown in the inset of Fig. 1d. The micro region X-ray photoelectron spectroscopy (XPS) measurement of the surface detects Pt with positive valance states and Te from PtTe_2_ simultaneously. The atomic ratio between Pt and Te is around 1 : 2 (Section S3). The migration of Cr to the surface after its substitution also reflects that the reaction has taken place thoroughly. From the EDS mapping of the Ge element in Fig. 1e, the Ge atoms disperse all over the vdW layers and form the layered structure in the framework of PtTe_2_, arising from the original layered distribution of Ge atoms in CGT. By overlapping the EDS mapping of the Ge element with the HAADF image in Fig. 1e, it is found that the EDS signal of Ge mostly appears around the Te atoms without segregation, as highlighted in the inset of Fig. 1e, indicating the uniform insertion of Ge atoms. Therefore, considering the zone axis of the HAADF image, the majority of Ge atoms are aligned with Te in the atomic column along 〈2${\rm{\overline{11}}}$0〉 crystallographic orientation (in hexagonal primitive cell). Note that due to the insertion of Ge, the lattice parameter for one vdW layer is calculated to be 0.42 nm, which is slightly larger than the 0.4 nm of stoichiometric PtTe_2_.

In contrast, the substitution process is complete and almost no Cr atom remains in the transformed vdW material (below the measurement accuracy of EDS). Through electron energy loss spectroscopy (EELS), the substituted Cr is found to crystallize dispersedly outside the vdW layers, as shown in Fig. 1f. At local positions on the surface (red square in Fig. 1f), the Cr element exhibits strong characteristic peaks around 584 eV with the appearance of the O element at 532 eV due to the easy oxidization of Cr. In contrast, in the EELS spectrum, which is the integral of EELS area mapping (Fig. S2) obtained from the vdW layers below the surface (blue square in Fig. 1), only a strong wide characteristic peak of the Te element is observed, indicating the absence of residual Cr in the PtTe_2_ structure. The results of the EELS area mapping and line mapping both demonstrate the complete phase transition (Fig. S2 and Table S1), which is also demonstrated through XPS measurements with depth analysis (Fig. S4). EDS measurements are also performed to characterize the substituted Cr, which is dispersed in segregated microcrystals outside the 2D layers without the formation of a film (Fig. S5). The XPS further illustrates the oxidization and dispersion of accumulated Cr on the surface (Fig. S3). Hence the complete phase transition of CGT/Pt through the thermal diffusion reaction is in line with our expectation. In accordance with the main framework of PtTe_2_, which is observed through the TEM and Raman measurements in Fig. 1, we call this novel vdW magnet Ge-embedded PtTe_2_. The atomic ratio between Te and embedded Ge is identified through the results of EDS mapping, and the chemical formula of the vdW magnet is defined as PtTe_2_Ge_1/3_. Another significant feature of the EELS spectrum of obtained PtTe_2_Ge_1/3_ is the absence of an O peak compared to the easily oxidized Cr microcrystals in Fig. 1f, after the cross-section TEM sample exposed in air. This feature primarily reflects the high oxidation resistance of PtTe_2_Ge_1/3_, which is consistent with our proposal and will be characterized in detail below. It is worth mentioning that the synthesis process has no requirements for the substrate and both the mechanical exfoliation and wet transfer processes [40] are highly feasible when it comes to transferring the samples from the Si/SiO_2_ substrate, which suggests the flexibility of this vdW material.

**Figure 2.**
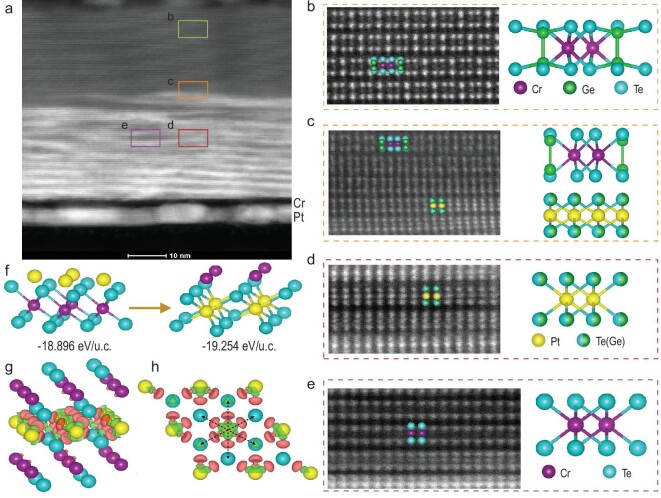
Structural characterizations of the 80 nm sample. (a) Low-resolution HAADF-STEM image of the cross section of the reacted *t* = 80 nm sample. Magnified HAADF-STEM images from the upper to lower regions, (b) the CGT layer, (c) transition area, (d) PtTe_2_Ge_1/3_ and (e) CrTe_2_. (f) Lattice structure variation and corresponding calculated energy for Pt replacing Cr, with the formation of PtTe_2_. DCD of Pt atoms between two layered CrTe_2_ from the (g) side view and (h) top view (isosurface value of 0.007 *e/*Bohr^3^). Red and green isosurface contours indicate the charge accumulation and reduction, respectively.

Limited by the amount of diffused Pt atoms, the phase transition to PtTe_2_Ge_1/3_ is incomplete in much thicker samples compared to the complete transition in thin samples, as demonstrated in Fig. 1. Therefore, the incomplete transition from CGT to PtTe_2_Ge_1/3_ in the thick sample can be employed to illustrate the detailed process of the thermal diffusion reaction. Figure 2a displays a low-resolution HAADF-STEM image of the cross section of the *t* = 80 nm sample. Obviously, there are two areas with distinct contrast in the vdW materials. The upper dark layer keeps pristine CGT without Pt due to the insufficient diffusion, as shown in Fig. 2b. Below this layer, a transition zone exists (Fig. 2c), where Ge-embedded PtTe_2_ emerges below the CGT layer, indicating the phase transition from CGT to Ge-embedded PtTe_2_. In the lower bright layer, the crystal is also not homogeneous, exhibiting both bright and dark parts. Based on the magnified images shown in Fig. 2d)and e, the bright and dark parts are found to be Ge-embedded PtTe_2_ and CrTe_2_, respectively. Because of the differences on both the space group and stoichiometry between CGT and PtTe_2_, the phase transition directly from CGT to PtTe_2_ is inaccessible. The observed CrTe_2_ bridges the phase transition between them.

We then address the question of how the phase transition occurs and present a plausible mechanism. During the thermal diffusion reaction, Pt atoms from the bottom layer diffuse into CGT and induce the decomposition of CGT to CrTe_2_ and GeTe, as described by the first step in reaction equation (1).

(1) \begin{eqnarray*} &&{\rm{CrGeT}}{{\rm{e}}}_{\rm{3}} + {\rm{Pt}} \to {\rm{CrT}}{{\rm{e}}}_{\rm{2}} \cdot \cdot \cdot {\rm{Pt + GeTe}}\\ &&\quad \to {\rm{PtT}}{{\rm{e}}}_{\rm{2}} \cdot \cdot \cdot {\rm{Cr + GeTe}} \to {\rm{PtT}}{{\rm{e}}}_{\rm{2}}{\rm{G}}{{\rm{e}}}_{{\rm{1/3}}}\\ \end{eqnarray*}

Through calculations of the total energy, we investigate the phase transition from CrTe_2_ to PtTe_2_. Corresponding data are shown in Fig. 2f. When Pt atoms are adjacent to CrTe_2_, the Pt atoms tend to immigrate into the CrTe_2_ layer and bond with Te atoms to reduce the total energy from –18.896 eV per unit cell (u.c.) to –19.254 eV/u.c., reflecting the energy preference to form PtTe_2_. This is shown by the second step in reaction equation (1). This process is bolstered by the calculation of the differential charge density (DCD), whose side view and top view are illustrated in Fig. 2g)and h, respectively. When Pt atoms are inserted between two CrTe_2_ vdW layers, an obvious charge redistribution occurs between Pt and interlayered Te. The charge accumulates around the *d* orbitals of Cr, near the interlayered Te, indicating that Pt atoms are bonded with the interlayered Te atoms and weaken the adjacent Cr–Te bonds. Therefore, Pt prefers to substitute Cr to form a Pt–Te octahedron, in which the HSAB principle [36] prevails over the metal activity sequence (Cr > Pt) and drives the phase transition. The substituted Cr atoms migrate from the vdW layer and crystallize at the bottom and top surfaces of the sample, as seen in Figs2a)and S5. Partial Ge atoms are embedded into PtTe_2_ [the third step in reaction equation (1)], and induce ferromagnetism. The rest of the Ge atoms volatilize in the form of small molecules of GeTe. As a result of the energy reduction and mobility of Pt atoms in CGT, the displacement reaction is theoretically able to complete itself with sufficient reaction conditions, such as enough Pt and reaction time. Nevertheless, according to the HAADF images shown in Fig. 2a)(Fig. S5), the Pt and vdW layer are separated at the bottom interface by spacers composed of Cr islands and void spaces, which originate from the substituted Cr and diffused Pt atoms, respectively. These spacers block the contact between Pt and the vdW layer and lead to the insufficiency of the diffusion reaction. The vdW layer can still stand on the substrate due to the discreteness of void spaces, with support from Cr islands.

**Figure 3.**
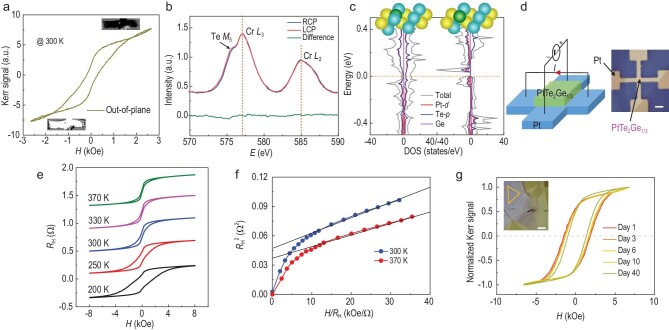
Magnetic characterizations and theoretical calculations. (a) Out-of-plane Kerr signal measured at 300 K. The insets show the images captured by MOKE under positive and negative magnetic fields. (b) Cr *L*_2,3_-edge X-ray absorption measurements performed using the right-circular polarized (RCP) and left-circular polarized (LCP) lights in an applied magnetic field of 4.5 kOe at 200 K; the corresponding difference of two XAS signals shows the absence of the XMCD of the Cr element. The relative intensity compared to the background is exhibited. (c) DOS calculations of Ge atoms located at the interstitial position between two Te atoms and the substitution position of Te atoms. Corresponding lattice structures are shown in the insets. Yellow, blue and green balls represent Pt, Te and Ge atoms, respectively. (d) Schematic of transport measurement set-up and an optical microscope image of the device. Scale bar: 10 μm. (e) *R*_H_–*H* of PtTe_2_Ge_1/3_ at representative temperatures from 200 to 370 K. (f) Arrott plots of PtTe_2_Ge_1/3_ at 300 and 370 K. (g) Kerr signals of the sample measured at room temperature after exposure in air for days. The inset exhibits the optical microscope image of the sample. Scale bar: 10 μm.

Then we check whether enhanced ferromagnetism exists in the brand-new phase, as proposed above. The magnetization hysteresis loop of obtained PtTe_2_Ge_1/3_ (∼10 nm), measured at room temperature (300 K) by the magneto-optical Kerr effect (MOKE) in Fig. 3a, undoubtedly characterizes ferromagnetism. Remarkably, the magnetization is still robust at room temperature, with considerable remanence (>45%), indicating a *T*_C_ above room temperature. The clear MOKE contrast at ± 2000 Oe displayed in the insets also supports the magnetization reversal and the ferromagnetism with the tendency of PMA at room temperature. The origin of the shape of the hysteresis loop will be discussed below. Note that the substituted Cr, dispersing in microcrystals on the surface, may confuse the origin of the room-temperature ferromagnetism. Thus, the measurement of X-ray magnetic circular dichroism (XMCD) [41,42], which is a sensitive element-specific probe of magnetic orders with surface sensitivity (detection depth ∼5 nm), was carried out. The result in Fig. 3b)exhibits the negligible difference between two X-ray absorption spectra (XAS) signals around Cr *L*_2,3_-edges [32,43], demonstrating the absence of its XMCD, which excludes any ferromagnetic order contributed by Cr in the state of crystals or other Cr compounds (such as Cr-oxides). Therefore, the ferromagnetism is contributed by the vdW PtTe_2_Ge_1/3_. We also notice a shoulder peak at 575 eV (2 eV lower than the Cr *L*_3_-edge), which most likely originates from the Te *M*_5_-edge [44,45].

The origin of ferromagnetism is then theoretically analyzed. Based on the analysis of the Ge element by EDS mapping in Fig. 1e, the Ge atoms are mostly aligned with Te in the atomic column, along 〈2${\rm{\overline{11}}}$0〉 crystallographic orientation. Therefore, there are two reasonable locations for the Ge atoms in PtTe_2_Ge_1/3_, consistent with the EDS result: the interstitial position between two adjacent Te atoms from the same atomic layer, and the substitution of Te atoms, as shown in the insets of Fig. 3c. Accordingly, we construct two structure models and calculate the spin-dependent density of states (DOS), as presented in Fig. 3c. Different from the pristine PtTe_2_ with spin conversed DOS (Fig. S6), a sizable ferromagnetism is generated in both structures and the ferromagnetism mainly originates from the 5*d*-electrons of Pt atoms and 5*p*-electrons of Te atoms. The introduction of the Ge atoms in PtTe_2_ not only slightly enlarges the lattice (Fig. 1b), but also breaks the symmetry and balanced state occupation of original Pt–Te octahedral fields, which induces magnetic moments on both Pt and Te atoms. In contrast, Ge atoms have little contribution to the magnetic moments, as demonstrated by the DOS in Fig. 3c. The electronic structures with not-fully-occupied orbitals generate magnetic moments of ∼0.8 *μ_B_* and ∼2.0 *μ_B_*, with the embedding of Ge atoms in the condition of insertion and substitution, respectively. Because of the uniform embedding of the Ge atoms with the layered structure and high density, exchange coupling exists among the generated magnetic moments, which is able to produce long-range ferromagnetic ordering. The *T*_C_ and magnetic anisotropy of the ferromagnetism are also enhanced by the strong spin-orbit coupling (SOC) [46] in PtTe_2_, which enables it to persist above room temperature with PMA [35,47]. The calculation of magnetocrystalline anisotropy energy (MAE) also reveals that the out-of-plane magnetic configuration is more stable with an energy that is ∼10.3 meV and ∼1.5 meV lower than the in-plane magnetic configuration per cell, in conditions of the interstitial position between Te atoms and the substitution of Te atoms, respectively (Table S3).

We further characterize the above-room-temperature ferromagnetism of PtTe_2_Ge_1/3_ in detail. Figure 3d is a schematic of the transport measurement set-up and an optical microscope image of the device. The out-of-plane magnetic-field-dependent anomalous Hall resistance (*R*_H_–H) curves of PtTe_2_Ge_1/3_ at different temperatures, from 200 K to 370 K, are shown in Fig. 3e. The most eminent feature is that a clear hysteresis loop with a large remanence at zero field persists up to 370 K (the highest temperature of our transport equipment), indicating a *T*_C_ far above room temperature. The Arrott plots in Fig. 3f, extracted from the representative *R*_H_*–H* loops, further demonstrate a *T*_C_ above 370 K. The gradual decrease of the slope in the Arrott plots indicates the attenuation of ferromagnetism from 300 K to 370 K due to thermal fluctuation. We emphasize that, distinct from conventional vdW magnets with magnetic element-based magnetism, the ferromagnetism induced in the present vdW magnets possesses high air stability. The EELS spectrum of the PtTe_2_Ge_1/3_ cross-section TEM sample (Fig. 1f), after being exposed in air, shows no signal of the O element, directly reflecting its high oxidation resistance. XPS data show the high stability (Fig. S4). Therefore, the ferromagnetic PtTe_2_Ge_1/3_ thin flakes (∼10 nm) are endowed with high air stability and show hardly any change in morphology and structure after being exposed in air for weeks, which is in stark contrast to the oxidization of conventional vdW magnetic CGT (Fig. S7). The substituted Cr in the form of segregated microcrystals, rather than a film on the surface (Figs S3 and S5), cannot prevent direct contact with oxygen from the top and side surfaces, which has no effect on air stability. Figure3g)shows the variation in magnetism of PtTe_2_Ge_1/3_ with time, measured by MOKE at room temperature without protection. Compared to the rapid oxidation in conventional vdW magnets, the sample shown in the inset exhibits stable ferromagnetism after 40 days, with negligible degradation and slight reduction of coercivity. This behavior is identical in other samples (Fig. S8). Compared to other vdW thin flake systems with air stability but a much lower *T*_C_, such as CrSe_2_/WSe_2_ with a *T*_C_ below 130 K [48], the above-room-temperature and air-stable ferromagnetism in present vdW PtTe_2_Ge_1/3_ advances the practical applications of vdW magnets.

**Figure 4.**
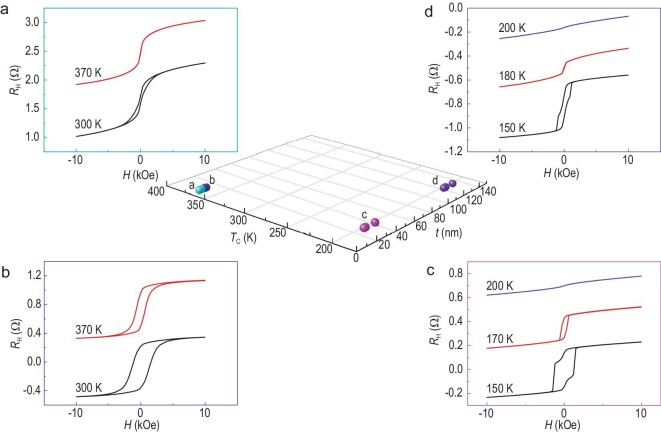
*R_H_*–*H* curves in the reacted CGT/Pt samples with different thicknesses. (a) *t* = 10.5 nm, (b) *t* = 14 nm, (c) *t* = 31 nm and (d) *t* = 125 nm.

Magnetism is also found to be relevant to thickness, and Fig. 4 shows the *T*_C_ as a function of the total sample thickness (*t*), which includes the Cr microcrystals on the surface. The enhancement of the *T*_C_ above room temperature can be observed in samples with a thickness from *t* ∼ 9 to *t* ∼ 17 nm, in which the *R*_H_*–H* results of *t* = 10.5 nm and *t* = 14 nm samples are representatively presented in Fig. 4a)and b, respectively (results of other samples are exhibited in Fig. S9). For the *t* = 10.5 nm sample, a clear hysteresis loop is obtained at *T* = 300 K, while the loop shrinks to a simple ‘*S*’ shape as the temperature increases to 370 K, which is very close to *T*_C_. Interestingly, a square loop indicating robust PMA exists in the *t* = 14 nm sample, way above room temperature (Fig. 4b), with remanence ∼80% at 300 K and remanence >60% at 370 K. The hysteresis loops with a ‘bee waist’ shape observed in the thinner samples of ∼10 nm (Fig. 3a)and e, Fig. 4a) are attributed to the weakened magnetic anisotropy and the dipolar interaction that generally exists in magnetic multilayers and vdW magnets, such as Co/Pd multilayers [49] and Cr_2_Ge_2_Te_6_ [13,50]. And the magnetic anisotropy is enhanced when the thickness increases and dominates the square shape of the hysteresis loops in thicker samples (Fig. 4b). The attenuation of magnetic anisotropy and *T*_C_ in thinner samples is due to the enhanced thermal fluctuation with decreased thickness, which is the characteristic of vdW magnetic thin flakes [7]. The variation of magnetic anisotropy with thickness, which is opposite to conventional magnetic films, also indicates that the origin of the room-temperature ferromagnetism is the vdW layered material PtTe_2_Ge_1/3_. When the thickness further increases to 30–40 nm, the *T*_C_ abruptly drops way below room temperature. Figure 4c illustrates the *R*_H_–*H* curves recorded at 150, 170 and 200 K of the *t* = 31 nm sample, which exhibits a *T*_C_ around 200 K. A similar behavior with a smaller coercivity and weaker PMA is observed in the *t* = 125 nm sample. Also visible in Fig. 4c)and d is the two-step switching at 150 K, which vanishes at higher temperatures. The separate switching indicates the coexistence of two phases in comparatively thicker samples; nevertheless, there is no magnetic coupling between the two phases (Fig. S10), and the proportion of the phase with weaker magnetic anisotropy increases with thickness.

The thickness-dependent ferromagnetism also reflects the process of thermal diffusion reactions. In samples with *t* ≤ 17 nm, the Pt atoms are able to diffuse to the whole vdW layer thoroughly, accompanied by the formation of a uniform PtTe_2_Ge_1/3_ with a *T*_C_ above room temperature. The thicker samples (*t* ≥ 30 nm) differ dramatically. Only the lower part of the CGT can be diffused by the Pt atoms, and the phase transition to the Ge-embedded PtTe_2_ phase only exists in the region adjacent to the Pt layer, while the upper part keeps the CGT phase unchanged. The two layers result in two-step switching (Fig. 4c)and d), in which the ferromagnetism with stronger PMA (larger *H*_C_) and higher *T*_C_ originates from the inhomogeneous transition part with the formation of discontinuous Ge-embedded PtTe_2_ phase. Due to the incomplete formation of Ge-embedded PtTe_2_ in the transition part diffused by Pt, its *T*_C_ is only ∼200 K, lower than that of uniform PtTe_2_Ge_1/3_, which is obtained in thinner samples. In contrast, the CGT part contributes to the weaker counterpart with a lower *T*_C_. This part plays a more important role in magnetization as the CGT thickness is increased, resulting, somehow, in the decay of ferromagnetism (Fig. 4d). According to the thickness-dependent ferromagnetism, the above-room-temperature ferromagnetism can also be attributed to the vdW PtTe_2_Ge_1/3_ (see detailed discussion in Section S10).

## CONCLUSION

In conclusion, given the difficulty of achieving room-temperature air-stable vdW magnets by conventional methods, we delicately obtained a novel 2D vdW magnet, Ge-embedded PtTe_2_ (PtTe_2_Ge_1/3_), through an anomalous displacement reaction driven by the HSAB principle. It shows robust ferromagnetism, combined with a *T*_C_ above room temperature, PMA and high air stability. The layered uniformly embedded Ge atoms result in long-range ordered ferromagnetism contributed by both Pt-5*d* and Te-5*p* electrons. Meanwhile, the compatibility of soft-acid Pt and soft-base Te ensures high air stability. Other methods of preparing similar ordered-doping vdW magnets, such as molecular beam epitaxy or chemical vapor deposition, are much anticipated. Our findings represent a significant step towards practical spintronics based on 2D vdW magnets and creates the potential to explore phase transitions between different vdW families.

## METHODS

### Sample preparation

Platinum was deposited on the Si/SiO_2_ substrate by magnetron sputtering, with a vacuum higher than 10^–^^7^ Torr. CGT flakes were then exfoliated using polydimethylsiloxane. No protective layer was deposited after the exfoliation of CGT. The heating process was also performed in the magnetron sputtering system with a vacuum higher than 10^–^^7^ Torr.

### Materials characterizations

Raman analysis was carried out using a HORIBA Raman microscope with an excitation wavelength of 532 nm. The thicknesses of the samples were measured by an atomic force microscope and the detailed results are shown in Fig. S11. Cross-section samples were fabricated by using a focused ion beam (FIB) system, with platinum deposited on the sample surface to prevent destruction by the ion beam. HAADF-STEM images, atomic resolved X-ray EDS and EELS spectra were performed on a FEI Titan Cubed Themis 18 60–300 (operated at 300 kV). Transport measurements were performed in a Quantum design physical property measurement system (PPMS). The Kerr signal and images of magnetic domains were captured by a MagVision Kerr Imaging System, which operates based on the MOKE in the polar configuration. The out-of-plane magnetization was probed and observed as different levels of brightness in the image. XAS of Cr *L*_2,3_-edge measurements were conducted in the total electron yield (TEY) detection mode using right-circular polarized (RCP) and left-circular polarized (LCP) lights in an applied magnetic field of 4.5 kOe at 200 K for the stronger signal, and vacuum pressure of 8 × 10^–^^8^ Torr at Beamline BL08U1A of the Shanghai Synchrotron Radiation Facility (SSRF). All spectra were obtained with the radiation normally incident to the film surface. The energy resolution of XAS was set to 0.2 eV, and the Cr *L*_2,3_-edge spectra were normalized to the maximum intensities of the Cr *L*_3_ peak. The binding energies in micro-region XPS measurements were corrected by referencing the measured C 1*s* peak to the value of 284.8 eV.

### Device fabrication

For magneto-transport, 5-μm-wide Hall bar devices were made by photolithography and ion milling.

### First-principle calculations

Our first-principle calculations were performed using the Vienna *ab initio* simulation package (VASP) [51,52] with projector augmented wave method [53,54]. The Perdew-Burke-Ernzerhof (PBE) functional [55] was used to treat the exchange correlation interaction and the plane-wave basis. The Gamma centered k-point mesh of 12 × 12 × 1 was used in all calculations without SOC. A vacuum layer larger than 15 Å was adopted in all calculations of thin films. DFT-D3 [56] was used to properly treat the interlayer vdW interaction. The DOS calculations of Ge-embedded PtTe_2_ and pristine PtTe_2_ were based on the same lattice constant, which was expanded to 0.46 nm for the insertion of Ge, as discussed in Fig. 3. The calculations of the total energy were also based on VASP. The MAE calculation was performed using the method GGA + SOC + *U* (*U*_eff_ = 3 eV for Pt [57,58]) with a Gamma centered k-point mesh of 7 × 7 × 1.

## Supplementary Material

nwac173_Supplemental_File
