# Supplementary material for: Anomalous displacement reaction for synthesizing above-room-temperature and air-stable vdW ferromagnet PtTe2Ge1/3
Source: Natl Sci Rev. 2022 Aug 18;10(1):nwac173. doi: 10.1093/nsr/nwac173 (PMC9843128; doi:10.1093/nsr/nwac173)
Supplement: nwac173_Supplemental_File [file nwac173_supplemental_file.pdf]

SUPPLEMENTARY MATERIALS

Anomalous displacement reaction for synthesizing above room-temperature and air-stable vdW ferromagnet $PtTe_2Ge_{1/3}$

Wenxuan Zhu, Cheng Song,* Qian Wang, Hua Bai, Siqi Yin, Feng Pan*

Key Laboratory of Advanced Materials, School of Materials Science and Engineering, Beijing Innovation Center for Future Chips, Tsinghua University, Beijing 100084, China

*Corresponding authors. E-mails: songcheng@mail.tsinghua.edu.cn; panf@mail.tsinghua.edu.cn

Section S1. Raman spectrum of exfoliated CGT.

The Raman spectrum of exfoliated CGT on Pt shows two characteristic modes of CGT, illustrating the good crystal quality (Fig. S1). The comparison between Fig. 1d in the main text and Fig. S1 demonstrates the formation of a new phased based on $PtTe_2$.

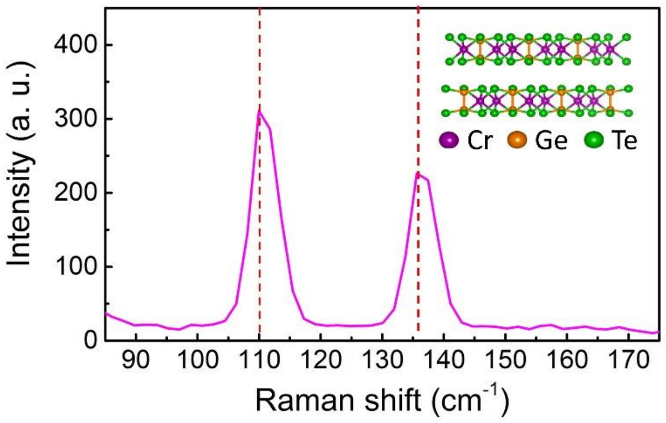

Figure S1. Raman spectrum of pristine CGT. The inset shows the atomic structure of CGT.

Section S2. EELS mapping of $PtTe_2Ge_{1/3}$.

The EELS mapping is performed to further demonstrate the complete phase transition. The results of the area mapping of large scale with the low resolution and local scale with the high resolution are shown in Fig. S2a–c and Fig. S2d–f, respectively. The whole $PtTe_2Ge_{1/3}$ region is homogeneous, filled with Te and without the appearance of Cr. The values of the signal strength and atomic ratios, obtained from the area mapping are shown in Table S1. The results demonstrate the absence of Cr in $PtTe_2Ge_{1/3}$. The line mapping is also performed at the bottom and top surfaces exhibited in Fig. S2g, h and Fig. S2i, j, respectively. The spectrum images of both surfaces adjacent to the crystallized Cr illustrate layered signal with obvious strength at Te peak and without the observation of Cr-$L_{2,3}$ edges, indicating the fully discharge of the Cr atoms. The integrated spectra of EELS line mapping are summarized in Fig. S2k. The Cr-$L_{2,3}$ and Te-$M_{4,5}$ edges shown in Fig. 1f were used for the acquisition of the spectrum maps.

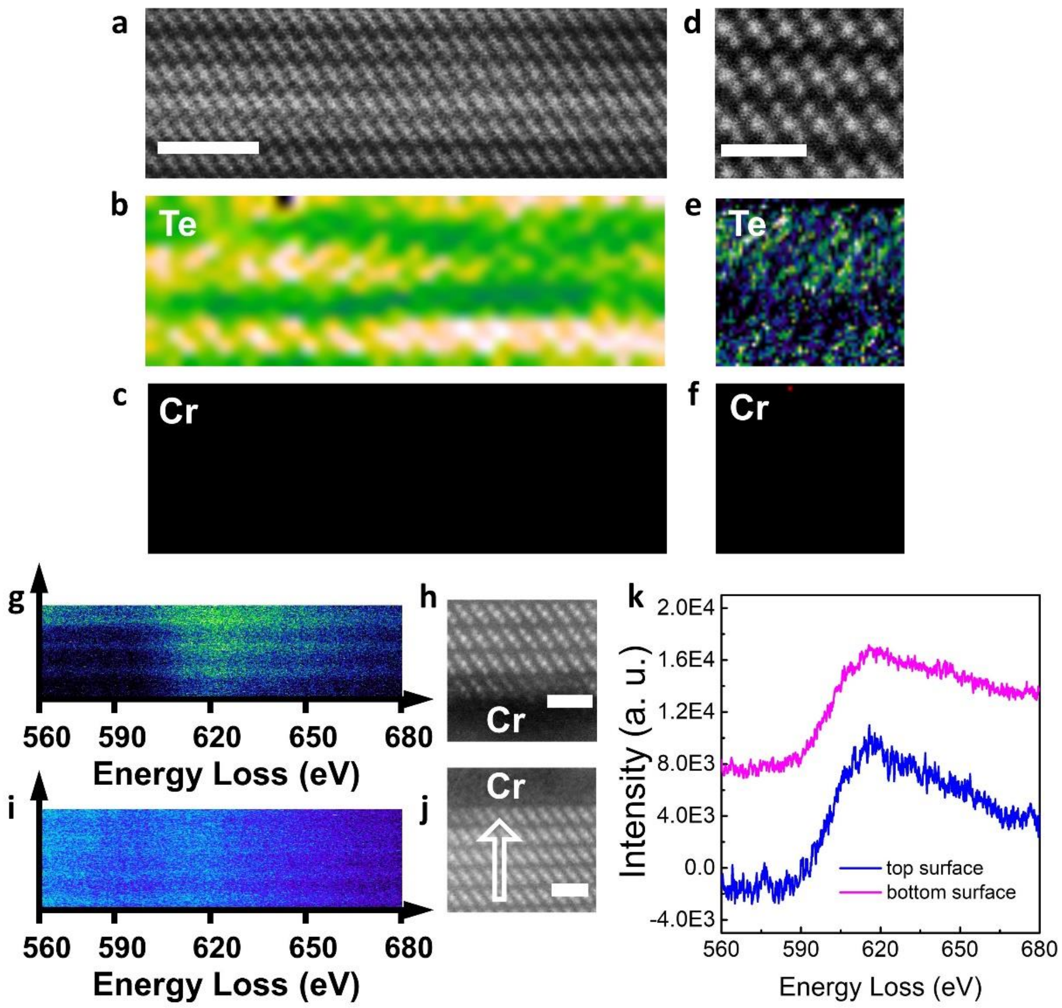

Figure S2. EELS mapping of $PtTe_2Ge_{1/3}$. HAADF, corresponding area mapping spectrum images of Te element and Cr element in the large scale with the low resolution (a–c) and local scale with the high resolution (d–f). Spectrum image and HAADF of the line mapping at the bottom surface (g, h) and top surface (i, j) adjacent to the crystallized Cr. (k) Integrated spectra according to the spectrum images in (g) and (i). Scale bar: 1 nm.

Table S1. Signal strength (counts) and atomic ratio (%) based on EELS area mapping of large scale/local scale.

Element	Signal strength	Atomic ratio
Te	1.4E8±2.7E4/9.7E7±2.2E4	1.0/1.0
Cr	0.0±2.7E4/0.0±2.2E4	0.0/0.0

Section S3. XPS characterization of the surface.

The micro-region XPS measurements were performed to characterize the surface after the thermal diffusion reaction. The XPS spectra of different elements are shown in Fig. S3. The signal of Pt from the substrate and the sample are detected simultaneously because the sample does not cover the full range of detection (radius of 20 μm). Based on XPS-peak-differentiating analysis of the asymmetric Pt characteristic peaks shown in Fig. S3a, the detection of Pt $4f_{7/2}$ and $4f_{5/2}$ peaks from the sample possesses higher energy than from the elementary Pt on substrate which demonstrates the electron loss of Pt and compound formation with Te. According to the surface sensitivity of XPS, the simultaneous detection of Pt with the positive valance state and Te on the surface with the atomic ratio of 1 : 2.5 shown in Fig. S3b (close to 1 : 2) indicates the diffusion of Pt up to the surface and already formed $PtTe_2$ structure on top of the sample reflecting the fully transition.

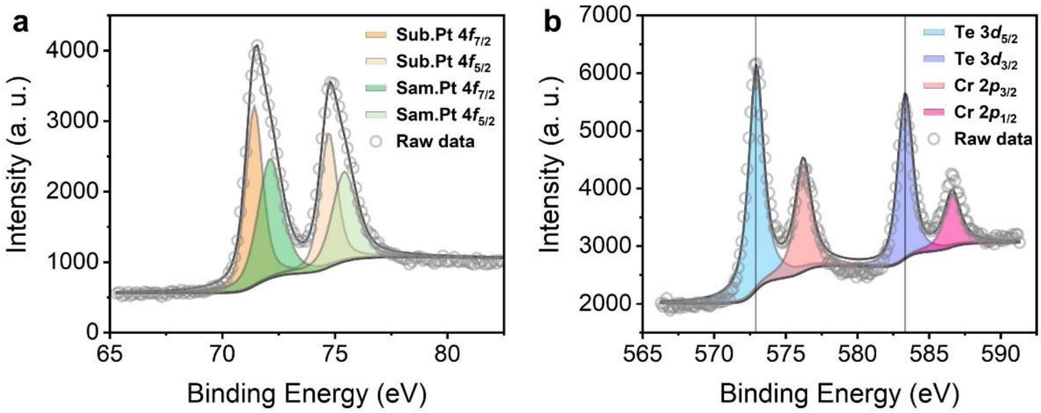

Figure S3. XPS spectra of the elements (a) Pt, (b) Te and Cr of the sample surface. The peaks of Te element are marked for comparison.

The characteristic peaks of Cr shown in Fig. S3b exhibit the valance state of +3 according to the $2p_{3/2}$ peak at 576.2 eV and $2p_{1/2}$ peak at 586.5 eV, which is due to the

easy oxidization of substituted Cr on the sample surface. Based on the XPS spectrum, the amount of Cr is even higher than Te on the surface with the atomic ratio of 1.3 : 1. The Cr atoms with major proportion on the surface results from the migration of Cr to the surface after substituted by Pt and suggests the transition which has already existed from the bottom to the top and leads to the accumulation of Cr on the top of vdW layers. After a short-time etching of the surface, the accumulated Cr on the surface is removed and the its signal is completely absent in the XPS spectrum shown in Fig. S4a. The existence of strong signal from Te with the disappearance of Cr in Fig. S4a demonstrates the fully substitution of Cr without residual in the vdW layer and the complete phase transition. The obviously detectable Pt, Te elements also reflect the Cr on the surface is segregated without the formation of a film.

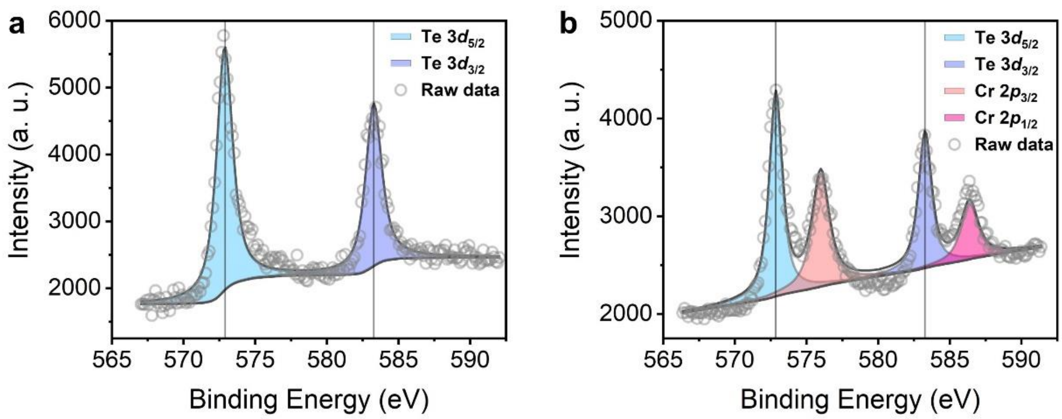

Figure S4. XPS spectra of the surface (a) with surface etching and (b) with exposure to air for ten days. The peaks of Te element are marked for comparison.

XPS measurements were also performed to characterize the stability. For comparison, Te XPS peaks after exposed to air for ten days is shown in Fig. S4b. No shift of Te peaks to the higher binding energy compared to the pristine surface signal in Fig. S3b and the bulk signal after etching in Fig. S4a illustrates the absence of its

electron loss and oxidization. Therefore, the combination of Pt and Te guarantees the high stability which is verified through the XPS measurements.

Section S4. Low-resolution HAADF-STEM images and characterizations of crystallized Cr.

In low-resolution images of both 14 nm $PtTe_2Ge_{1/3}$ (shown in Fig. 1 in main text) and 80 nm sample (shown in Fig. 2 in main text), crystallized Cr is found at the bottom and top surfaces of samples as shown in Fig. S5a and b, respectively, which illustrates the whereabouts of the substituted Cr atoms. The top bright layer in the images is the protective layer for the preparation of STEM samples. Both of the EDS area mapping (Fig. S5c) and EELS (Fig. S5d) were performed to characterize the crystallized Cr. In EDS area mapping, it clear illustrates that only the Cr element is found in the dark area squared in red, barely no other element appears with Cr, demonstrating the formation of crystallized Cr rather than Cr compounds. The EELS also shows the similar results. The Cr element exhibits strong characteristic peaks around 584 eV, only accompanied by the appearance of O element at 532 eV owing to the easy oxidization of crystallized Cr. During the phase transition, due to the small atomic radius, Cr atoms tend to be discharged along the interlayer vdW gaps or the out-of-plane direction to reduce the total energy, which crystallized around the edges of samples. According to the inhomogeneous contrast as shown in Fig. S5e, the substituted Cr forms segregated microcrystals at the top and bottom surfaces instead of a continuous film, which exposes the surface of $PtTe_2Ge_{1/3}$.

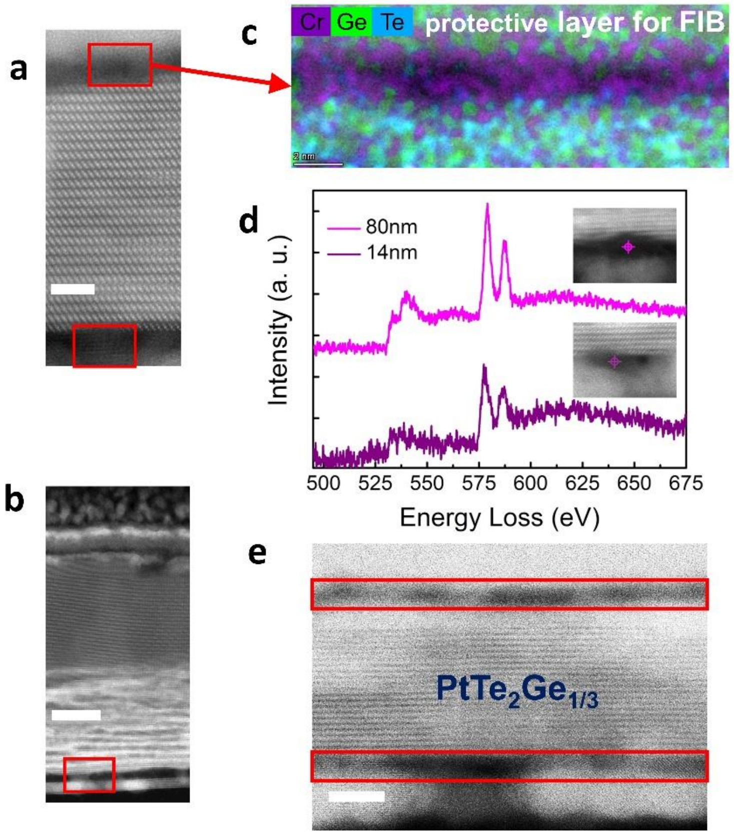

Figure S5. Characterizations of crystallized Cr atoms at the bottom and top surfaces of samples. Low-resolution HAADF-STEM images of 14 nm $PtTe_2Ge_{1/3}$ (Scale bar: 2.5 nm) (a) and 80 nm sample (Scale bar: 20 nm) (b). Cr atoms crystallize at bottom and top surfaces of samples (dark areas), which are framed in red. (c) EDS area mapping of Cr, Ge, Te elements in the dark area. The top layer is the protective layer for sample preparation by FIB. (d) EELS of the dark area. The double peaks around 584 eV are features of Cr element. The appearance of the peak of O element at 532 eV is due to the oxidation of Cr crystals during the preparation of samples. (e) Low-resolution HAADF-STEM image of 14 nm $PtTe_2Ge_{1/3}$ (Scale bar: 5 nm). The segregation of Cr microcrystals at the surfaces results in the inhomogeneous contrast, which is squared in red.

Section S5. Atomic Fraction based on EDS area mapping.

The atomic ratio between Te and Ge in the vdW magnet $PtTe_2Ge_{1/3}$ is obtained based on the EDS area mapping shown in Table S2. Due to the reason that the amorphous Pt protection layer of FIB will splash onto the cross section, the value of Pt based on EDS mapping is not the intrinsic information of the material and has no reference value, which is not shown in Table S2. Therefore, the ratio of atomic fraction between Te and Ge, rather than the absolute value, is used to characterize the composition.

Table S2. Atomic Fraction of Ge and Te elements based on EDS area mapping.

Element	Atomic Fraction (%)	Atomic Error (%)
Te	43.27	7.45
Ge	7.04	1.31

Section S6. DOS calculation of $PtTe_2$.

We also calculate the DOS of $PtTe_2$ without the insertion of Ge atoms to illustrate the origin of magnetism in $PtTe_2Ge_{1/3}$. As shown in Fig. S6, DOS of $PtTe_2$ based on the same lattice constant as Fig. 3c in the main text, shows no difference between the spin down and spin up, indicating the absence of ferromagnetism. Therefore, Ge is the key to induce ferromagnetism.

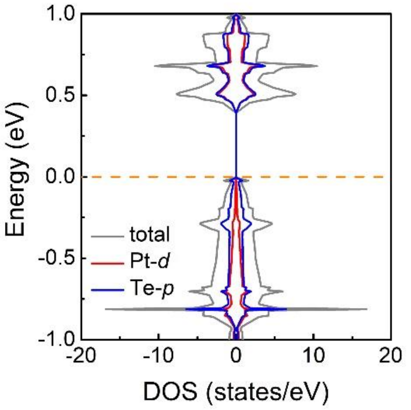

Figure S6. DOS of $PtTe_2$ without the insertion of Ge, based on the same lattice constant as Fig. 3c in the main text.

Table S3. Total energy of different magnetic orientations for MAE in two models (unit in eV). The stable ones are highlighted in blue.

orientation	Ge between Te	Ge substitutes Te
in-plane	−34.0354	−29.5704
out-of-plane	−34.0458	−29.5719

Section S7. Air-stability of $PtTe_2Ge_{1/3}$ and CGT.

To investigate the air stability of $PtTe_2Ge_{1/3}$, the sample was exposed to air for two months. The Raman spectra (Fig. S7a) and morphology (Fig. S7c and d) exhibits barely no change after exposed in air, demonstrating the high stability of $PtTe_2Ge_{1/3}$. In contrast, the Raman spectrum of CGT exhibits the change of characteristic peaks after exposed in air as shown in Fig. S7b, indicating the structural variation due to oxidization and the obvious depravation on morphology is observed after exposed in air for weeks (Fig. S7e and f). After exposed in air for longer time (two months), the morphology of CGT is severely damaged in Fig. S7g.

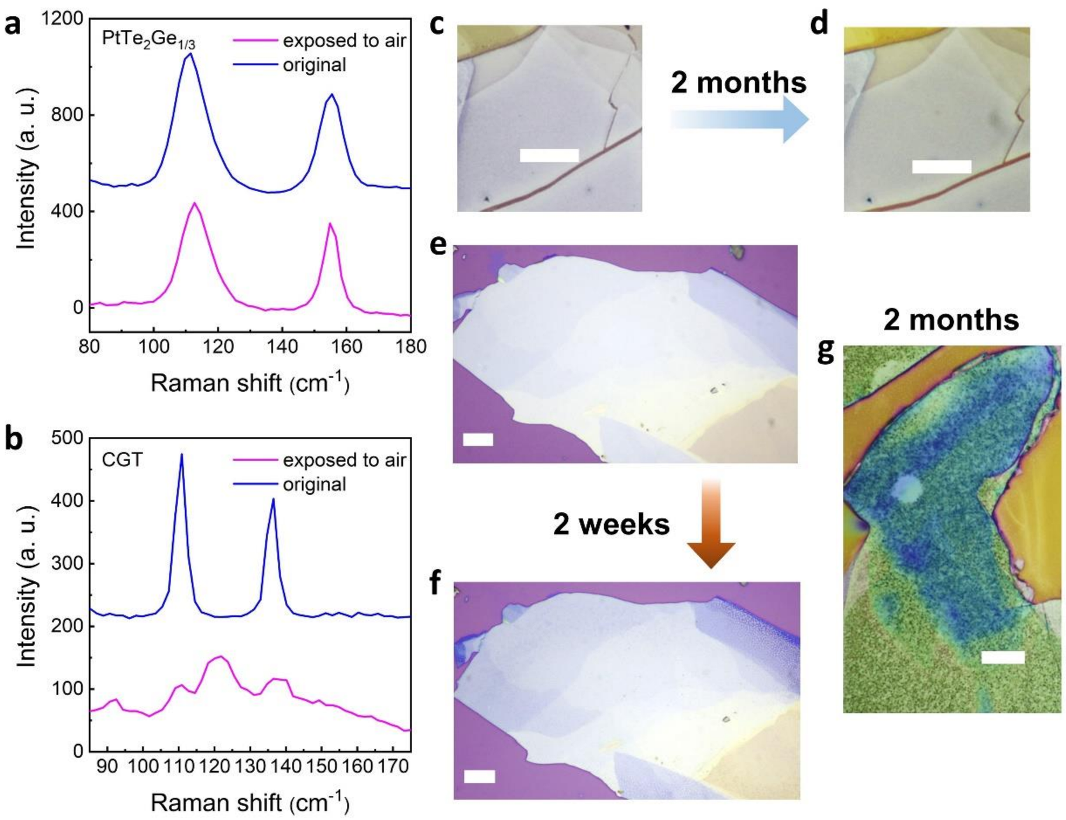

Figure S7. Time-variation of structure and morphology in air without protection. (a, b) Raman spectra of $PtTe_2Ge_{1/3}$ (a) and CGT (b) after exposed in air. Optical microscope images of $PtTe_2Ge_{1/3}$ (c) without exposure to air and (d) with exposure in air for 2 months. (e–g) Optical microscope images of CGT without exposure to air (e) and

exposure in air for 2 weeks (f) and 2 months (g). Scale bar: 10 μm.

In terms of the air stability of ferromagnetism, two more samples were measured besides the one shown in Fig. 3g in main text. All of them show the same high air stability on ferromagnetism demonstrated by the MOKE measurement shown in Fig. S8a and b, only with slight reduction of the coercivity (Fig. S8c).

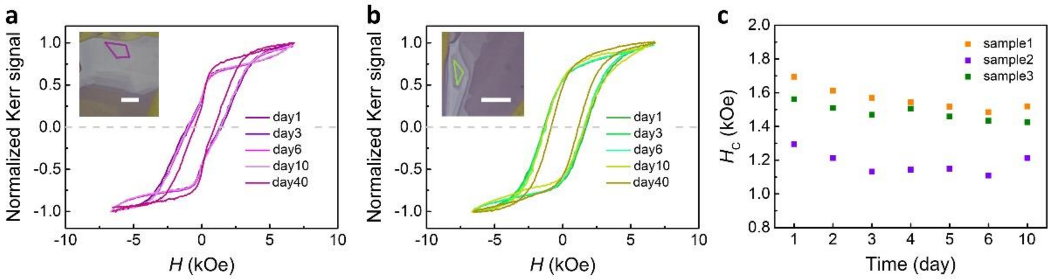

Figure S8. Time-variation of magnetism in air without protection. (a, b) Kerr signal of the two samples measured at room temperature after exposed in air for days. The insets exhibit the optical microscope images of the samples. Scale bar: 10 μm. (c) Variation of H_C with time of exposure to air. The samples shown in Fig. 3g in the main text, Fig. S8a and Fig. S8b are named as sample 1, sample 2 and sample 3, respectively.

Section S8. Characterization of magnetism in ~9 nm and ~17 nm samples

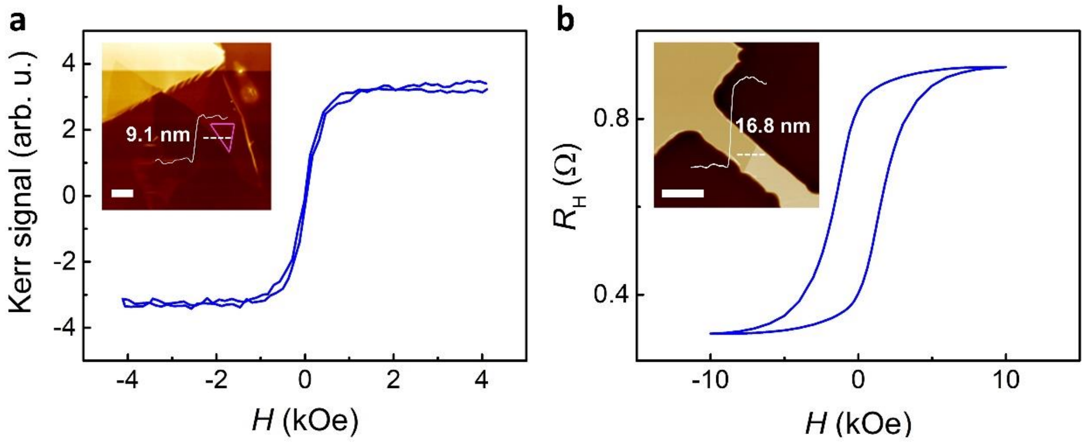

Figure S9. Ferromagnetism of ~9 nm sample (a) characterized by MOKE and ~17 nm sample (b) by the anomalous Hall effect at room temperature. The insets show the atomic force microscope images and height profiles. The dashed lines represent the paths of height profile and the magenta triangle (a) denotes the area where the Kerr signal is obtained. Scale bar: 5 μm.

Section S9. Minor loop of 31 nm sample at 150 K.

Due to the two-step switching of the 31 nm sample at 150 K (Fig. 4c in main text), we measure the minor loop to investigate the magnetic coupling between two phases. As shown in Fig. S10, the minor loop shows no shift indicating the absence of coupling between two phases.

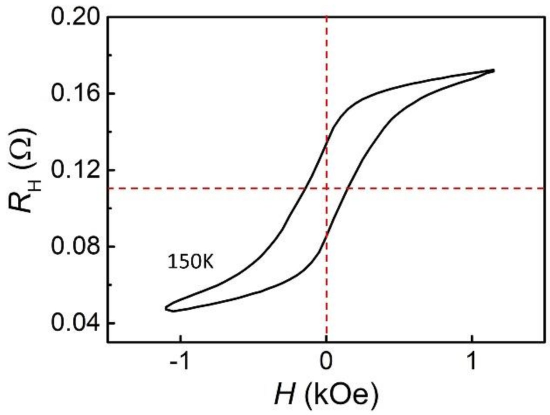

Figure S10. Minor loop at 150 K of Fig. 4c in the main text.

Section S10. Discussion on thickness-dependent ferromagnetism.

With careful analysis, we can also exclude the contribution of Cr to the room-temperature magnetism from the thickness-dependent ferromagnetism. From the analysis of HAADF-STEM, it is clear to see that the similar crystallized Cr exists in both of the uniform $PtTe_2Ge_{1/3}$ samples and thicker samples (Fig. S5). However, the ferromagnetism exhibits obvious variation with the sample thickness (Fig. 4 in the main text). In samples below 17 nm, the uniform new phase $PtTe_2Ge_{1/3}$ with barely no residual Cr is formed and possesses T_C above room temperature. In contrast, in thicker samples, more unsubstituted Cr-Te phases remain and T_C below 200 K instead, which rules out the possibility that Cr or Cr-Te compounds contribute to the room-temperature ferromagnetism. Especially in samples below 17 nm, which are able to form uniform $PtTe_2Ge_{1/3}$ and possess room-temperature magnetism, the magnetic anisotropy weakens with the decrease of thickness, which is the characteristic of 2D magnetism. Therefore, the contribution of middle phases or interfacial effects to the room-temperature magnetism is able to be excluded.

Section S11. Atomic force microscope image and height profile.

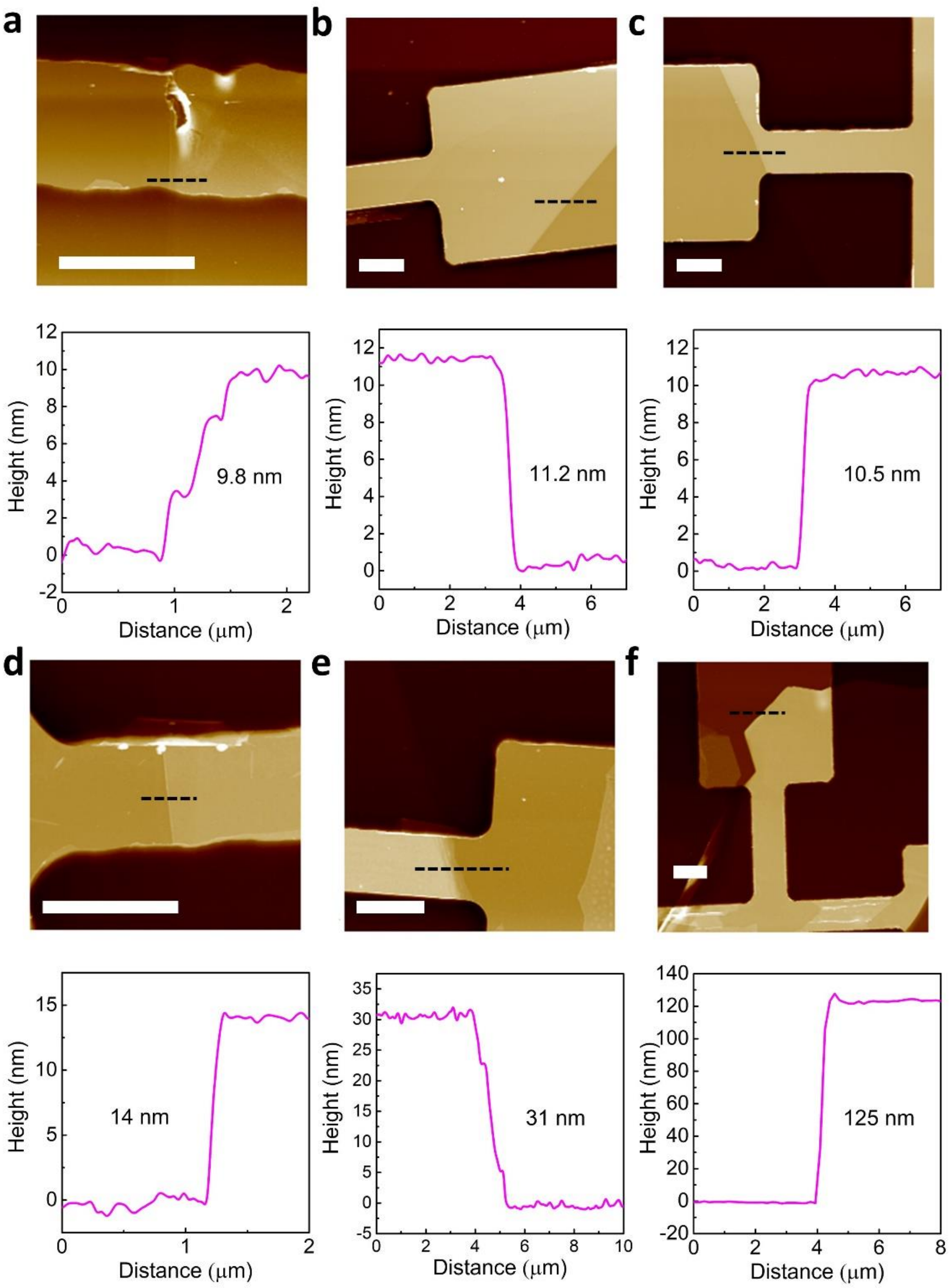

Figure S11. Atomic force microscope image and height profile of the sample in Fig. 3a (a), Fig. 3e (b) and Fig. 4a–d (c–f). The dashed lines represent the paths of the height profile. Scale bar: 5 μm.
